# Pre‐steady‐state kinetics and solvent isotope effects support the “billiard‐type” transport mechanism in Na ^+^‐translocating pyrophosphatase

**DOI:** 10.1002/pro.4394

**Published:** 2022-08-25

**Authors:** Anssi M. Malinen, Viktor A. Anashkin, Victor N. Orlov, Alexander V. Bogachev, Reijo Lahti, Alexander A. Baykov

**Affiliations:** ^1^ Department of Life Technologies University of Turku Turku Finland; ^2^ Belozersky Institute of Physico‐Chemical Biology Lomonosov Moscow State University Moscow Russia

**Keywords:** “billiard‐type” transport, energy coupling, membrane pyrophosphatase, Na^+^ transport, pre‐steady‐state kinetics, proton inventory, quenched flow

## Abstract

Membrane‐bound pyrophosphatase (mPPase) found in microbes and plants is a membrane H^+^ pump that transports the H^+^ ion generated in coupled pyrophosphate hydrolysis out of the cytoplasm. Certain bacterial and archaeal mPPases can in parallel transport Na^+^ via a hypothetical “billiard‐type” mechanism, also involving the hydrolysis‐generated proton. Here, we present the functional evidence supporting this coupling mechanism. Rapid‐quench and pulse‐chase measurements with [^32^P]pyrophosphate indicated that the chemical step (pyrophosphate hydrolysis) is rate‐limiting in mPPase catalysis and is preceded by a fast isomerization of the enzyme‐substrate complex. Na^+^, whose binding is a prerequisite for the hydrolysis step, is not required for substrate binding. Replacement of H_2_O with D_2_O decreased the rates of pyrophosphate hydrolysis by both Na^+^‐ and H^+^‐transporting bacterial mPPases, the effect being more significant than with a non‐transporting soluble pyrophosphatase. We also show that the Na^+^‐pumping mPPase of *Thermotoga maritima* resembles other dimeric mPPases in demonstrating negative kinetic cooperativity and the requirement for general acid catalysis. The findings point to a crucial role for the hydrolysis‐generated proton both in H^+^‐pumping and Na^+^‐pumping by mPPases.

## 1 INTRODUCTION

Membrane pyrophosphatases (mPPases, EC 7.1.3.1, formerly 3.6.1.1) are integral membrane proteins commonly found in the cytoplasmic membrane of diverse bacteria and archaea, the vacuolar membrane of plants, and the acidocalcisomal membrane of protozoa. mPPases hydrolyze PP_i_, a ubiquitous metabolic byproduct, to build up cation gradients in the cell, in contrast to soluble PPases, which dissipate PP_i_ energy as heat. All initially described mPPases couple pyrophosphate (PP_i_) hydrolysis to H^+^ pumping (H^+^‐PPases),^1^, ^2^ but later studies have identified an evolutionarily related prokaryotic subfamily that pumps Na^+^ (Na^+^‐PPases).^3^, ^4^, ^5^ Regardless of the subcellular localization, the direction of H^+^ and Na^+^ transport by mPPase is away from the cytoplasm. Bacterial H^+^‐PPases are essential for growth in energy‐limiting conditions.^6^, ^7^ Accordingly, plants overexpressing H^+^‐PPase are more tolerant to various stresses, such as drought, salinity, and nutrient limitation.^8^, ^9^ A similar biotechnological potential is expected for Na^+^‐PPase.^10^

Membrane PPases bear no sequence homology to other protein families. Both transport and hydrolytic activities associate with a single polypeptide made of 600–800 amino acid residues^11^, ^12^, ^13^ that form 15–17 transmembrane helices.^14^ The polypeptide functions as a homodimer,^15^, ^16^, ^17^, ^18^, ^19^, ^20^ whose constituent subunits exhibit kinetic and binding cooperativity.^21^, ^22^, ^23^

Membrane PPases resemble all other PPases in being Mg^2+^‐dependent enzymes, but some H^+^‐PPases and all Na^+^‐PPases additionally require K^+^ for maximal activity.^24^, ^25^, ^26^, ^27^ All K^+^‐dependent mPPases contain Ala in the last position of the GNXX(K/A) signature sequence.^21^, ^28^ While K^+^ usually acts as a modulator in the K^+^‐dependent mPPase, Na^+^, the coupling ion, is absolutely required for the hydrolytic activity of Na^+^‐PPases.^3^, ^29^ mPPase is also similar to other PPases in that PP_i_ hydrolysis proceeds as a direct attack of a water molecule on a phosphorous atom without the formation of a covalent intermediate.

The mechanism by which mPPases couple PP_i_ hydrolysis to cation transport has been controversial. Critical issues are the sequence of the hydrolysis and ion translocation events and the role of the hydrogen ion generated during the water attack on PP_i_. The mechanism first suggested for H^+^‐PPase based on its crystal structure^19^ implies that the transported H^+^ ion is the one generated from the water nucleophile (“direct coupling”) and, consequently, PP_i_ hydrolysis precedes the transport event. An alternative proposal, solely grounded in the data measuring the generation of transmembrane electric potential difference upon substrate analog binding,^30^, ^31^ was that H^+^ transport accompanies PP_i_ binding and precedes PP_i_ hydrolysis. However, this interpretation of the electrometric data was refuted later.^32^

Even less is known about the coupling mechanism in Na^+^ transport. All Na^+^‐PPases can catalyze a parallel translocation of Na^+^ and H^+^ at sub‐physiological Na^+^ concentrations (<5 mM),^33^ and some Na^+^‐PPases can simultaneously perform both transport functions even at excessive (up to 100 mM) Na^+^ concentrations.^34^ To explain the interplay between Na^+^ and H^+^ transport, we have suggested a “billiard‐type” mechanism, in which the proton released from the nucleophilic water molecule pushes pre‐bound Na^+^ through the ion conductance channel of Na^+^‐PPase.^26^, ^32^ This speculative mechanism extends the “direct‐coupling” H^+^‐PPase mechanism to Na^+^‐PPase.

This paper describes the first kinetic evidence favoring the billiard‐type mechanism in Na^+^‐PPases. Specifically, we identify the chemical step as rate‐limiting and Na^+^‐independent in PP_i_ hydrolysis and show that this step involves proton transfer in both H^+^‐PPase and Na^+^‐PPases. The experiments involving a rapid mixing step, which imposed limitations on enzyme stability, were performed with a highly stable *Thermotoga maritima* mPPase (Tm‐mPPase). We also present relevant kinetic characteristics of this enzyme.

## 2 RESULTS

### 2.1 Steady‐state kinetics of PP_i_ hydrolysis by Tm‐mPPase

Previous studies have detected negative kinetic cooperativity in PP_i_ hydrolysis and H^+^ transport by different mesophylic mPPases.^21^, ^23^ Specifically, substrate binding to one active site dramatically increased the Michaelis constant (K‐type cooperativity) and moderately decreased the other site's catalytic constant (V‐type cooperativity). These findings indicated that only one active site is predominantly operating in mPPase at any given time under in vivo conditions (substrate, metal cofactor, and H^+^ concentrations). The steady‐state kinetic measurements described below indicated similar behavior for Na^+^ transporting Tm‐mPPase. This mPPase, originating from the most thermostable bacterium, was used in rapid kinetic studies because of its high stability during the associated manipulations.

**FIGURE A1:**
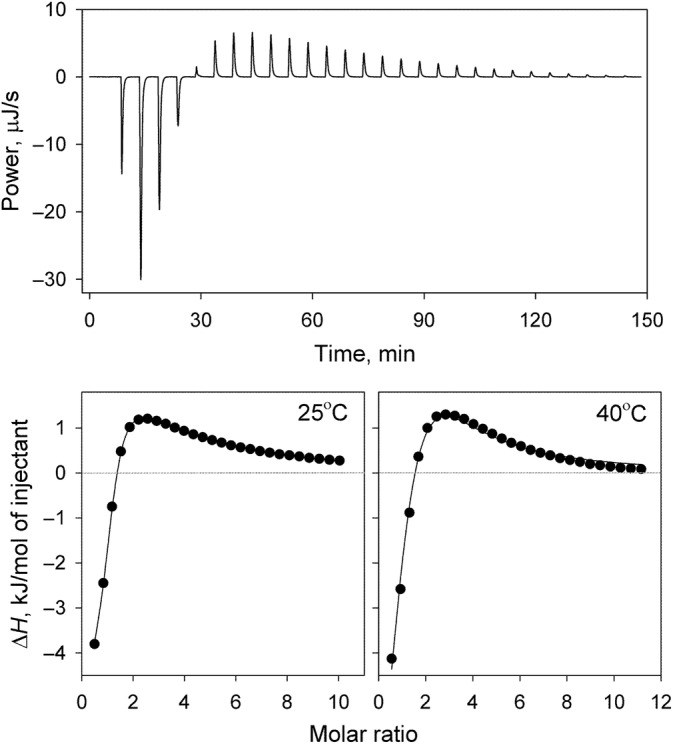
ITC measurements of magnesium binding to PP_i_. *Top*, typical raw data for successive injections of MgCl_2_ into 0.5 mM PP_i_ solution at 25°C after correction for dilution. *Bottom*, typical dependencies of integrated heats in PP_i_ titration by MgCl_2_ at 25 and 40°C. The lines are the best fits of a “sequential binding” model for two sites

The optimal temperature for Tm‐mPPase is 70–75°C, but our studies were conducted at 40°C, the temperature at which it is still reasonably active (15% of the maximum activity).^25^ This temperature choice, dictated in part by the phase instability of substrate solutions at higher temperatures, necessitated a reassessment of the stability constants for the magnesium pyrophosphate complexes, required to maintain constant free Mg^2+^ concentration and thereby make substrate (Mg_2_PP_i_) concentration a single factor affecting catalysis. We used isothermal titration calorimetry to determine these constants at 40°C (Table A1) (Appendix A, Figure A1). We also estimated Mg_2_PP_i_ solubility, which decreases with temperature and is another obstacle in kinetic measurements. Sedimentation analysis indicated that Mg_2_PP_i_ remains soluble to 800 μM at 40°C during the enzyme assay (Appendix A).

**FIGURE 1:**
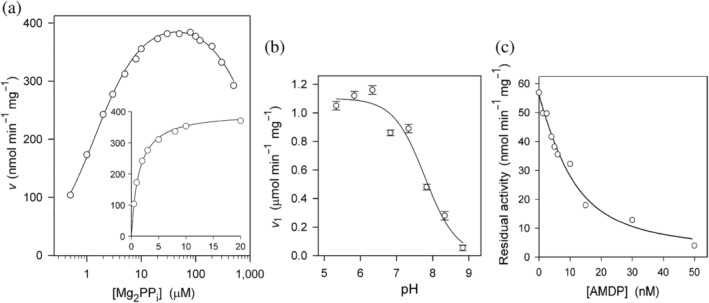
Steady‐state kinetics of Tm‐mPPase. (a) Substrate concentration dependence of Tm‐mPPase activity in IMVs at 40°C (0.1 M MOPS‐KOH, pH 7.2, 5 mM Mg^2+^, 10 mM NaCl). The theoretical curve was calculated with Equation (1) using the parameter values found in Table 1. The inset with a linearly scaled abscissa shows the ascending part of the curve. (b) The pH‐dependence of the maximal velocity for partially purified Tm‐mPPase at 40°C, as estimated from the ascending part of the substrate concentration dependence (0.5–100 μM Mg_2_PP_i_ range). The assay medium contained 20 mM Mg^2+^, 100 mM KCl, 0.2 mM KF, 0.1 mM EGTA, 3–100 mM NaCl, and 0.038 M each of MES, TES, TAPS, and CAPSO buffers adjusted to the desired pH with tetramethylammonium hydroxide. Bars refer to standard errors in the fitted *V*_1_ values. The theoretical curve was obtained with Equation (2) using the best‐fit p*K*_a_ value of 7.8. (c) A typical titration of the active sites of Tm‐mPPase in IMV. Steady‐state rates of 8 μM PP_i_ hydrolysis were measured at 25°C in the presence of 100 mM NaCl (no KCl added) at a varied concentration of AMDP, a tightly bound PP_i_ analog. The total IMV protein concentration was 0.02 mg ml^−1^. The solid line shows the best fit of Equation (3) with *K*_i_^app^ = 5 ± 2 nM and [E] = 10 ± 4 nM. AMDP, aminomethylene diphosphonate; CAPSO, 3‐(cyclohexylamino)‐2‐hydroxy‐1‐propanesulfonic acid; IMV, inverted membrane vesicle; MES, 2‐(N‐morpholino)ethanesulfonic acid; MOPS, 3‐(N‐morpholino)propanesulfonic acid; TAPS, N‐[tris(hydroxymethyl)‐methyl]3‐aminopropanesulfonic acid; TES, N‐[tris(hydroxymethyl)‐methyl]2‐aminoethanesulfonic acid; TMA, tetramethylammonium

The substrate saturation curve for Tm‐mPPase measured at 40°C in the presence of 5 mM Mg^2+^ (Figure 1a) demonstrated inhibition by excess substrate, in agreement with the data reported by Goldman's group^22^ but differed from them in demonstrating no sigmoidicity (positive substrate‐binding cooperativity in terms of *K*_m_) in the ascending part. We believe our data are more accurate at micromolar substrate concentrations due to the much greater sensitivity of our phosphate assay.^35^ A nearly identical substrate saturation curve was obtained at a 1 mM Mg^2+^ concentration.

These rate data were analyzed with Equation (1) derived for a homodimeric enzyme with two active sites^23^:

(1) $$v=\frac{\left(V_{1}+V_{2}\left[S\right]/K_{m2}\right)}{\left(1+K_{m1}/\left[S\right]+\left[S\right]/K_{m2}\right)}\;,$$

In this equation, S is the substrate (Mg_2_PP_i_), *K*_m1_ and *K*_m2_ are macroscopic Michaelis constants, and *V*_1_ and *V*_2_ are the specific activities of the mono‐ and di‐substrate enzyme species. Table 1 lists the parameter values derived with Equation (1) from Figure 1a and similar data measured at a 1 mM Mg^2+^ concentration. The *V*_2_ value was indistinguishable from zero within the error of determination, which was relatively high because the limited substrate solubility did not permit substrate concentrations significantly exceeding *K*_m2_. To reiterate, homodimeric Tm‐mPPase resembles other investigated mPPases in demonstrating increased *K*_m_ and decreased *k*_cat_ values for the second active site.^21^, ^23^ Because of the profound difference in the *K*_m_ values for two active sites in the mPPase dimer, the substrate predominantly occupies only one of them at <0.1 mM substrate concentrations.

**TABLE 1:** Parameters of Equation (1) for Tm‐mPPase‐catalyzed Mg_2_PP_i_ hydrolysis at two fixed Mg^2+^ concentrations

Parameter	Value	
	1 mM Mg^2+^	5 mM Mg^2+^
*V*_1_ (nmol min^−1^ mg^−1^)	396 ± 5	400 ± 5
*V*_*2*_ (nmol min^−1^ mg^−1^)	~0	~0
*K*_m1_ (μM)	1.48 ± 0.07	1.41 ± 0.04
*K*_m2_ (μM)	1,300 ± 100	1,400 ± 100

The rates of PP_i_ hydrolysis by Tm‐mPPase were also measured over the pH range of 5.5–9 at 0.5–100 μM Mg_2_PP_i_ concentrations to determine the *V*_1_ and *K*_m1_ values. These experiments were conducted at 40°C. A partially purified enzyme was used to exclude the membrane effect, and care was taken to ensure that the Na^+^ concentration was saturating and not inhibitory in all cases (higher Na^+^ concentrations were used at low pH values). The pH dependence of *V*_1_ (Figure 1b) revealed the requirement for a protonated group, and fitting Equation (2) determined its p*K*_a_ of 7.8 ± 0.1. The *K*_m_ value varied only twofold in this pH range, indicating that the p*K*_a_ of this ionizable group is similar in the substrate‐free enzyme and enzyme‐substrate complex, which means that the group does not interact with the substrate directly. Similar general acid groups were found in *Methanosarcina mazei* mPPase (Mm‐mPPase) (p*K*_a_ = 9.3)^36^ and *Vigna radiata* mPPase (p*K*_a_ = 8.6).^37^ The protonated group is unlikely to be part of the transport machinery because soluble non‐transporting PPases demonstrate a similar dependence on general acid catalysis.^38^ Neither this group belongs to the nucleophilic water because deprotonation would activate it.

(2) $$V_{1}=\frac{V_{1,\lim}}{\left(1+10^{\mathrm{pH}-{pK}_{a}}\right)},$$

Analysis of pre‐steady‐state enzyme kinetics described below required knowledge of active site concentration, which should be comparable with the concentration of the reaction product. We could determine the Tm‐mPPase concentration in inverted membrane vesicle (IMV) due to the extremely high affinity of the enzyme for the non‐hydrolyzable PP_i_ analog aminomethylene diphosphonate (AMDP), which acts as a competitive inhibitor.^23^ The true binding constant for AMDP was as low as 1.5 nM, allowing the titration of the Tm‐mPPase active sites by measuring enzyme activity at similar enzyme and AMDP concentrations at a non‐saturating substrate concentration. The titrations were performed at 25°C without K^+^ to decrease enzyme activity, thereby allowing a higher enzyme concentration in the assay.

A typical dose‐dependence curve is shown in Figure 1c. The inhibition data were analyzed with Equation (3)^39^ derived by solving the quadratic mass‐balance equation. Here, *v*_i_ and *v*_o_ are the inhibited and non‐inhibited rates, respectively, and [I] is the AMDP concentration. Fitting Equation (3) to the dependence of the rate of the mPPase reaction on [I] allowed the estimation of the apparent inhibition constant for AMDP (*K*_i_^app^) and binding site concentration ([E]) treated as adjustable parameters. The determined [E] value in the stock IMV (8–10 μM in terms of the dimer, depending on the IMV preparation) was sufficient to assess pre‐steady‐state kinetics with the quenched‐flow method. The error in [E] was relatively high because the competing substrate, present in the assay medium, increased the inhibition constant, *K*_i_^app^, to ~5 nM.

(3) $$\frac{v_{i}}{v_{0}}=1-\frac{\left\{\left[E\right]+\left[I\right]+K_{i}^{\mathrm{app}}-\sqrt{{\left(\left[E\right]+\left[I\right]+K_{i}^{\mathrm{app}}\right)}^{2}-4\left[E\right]\left[I\right]}\right\}}{2\left[E\right]},$$

Based on Tm‐mPPase subunit mass of 77 kDa and assuming one tight binding site for AMDP per enzyme dimer,^23^ Tm‐mPPase accounted for 7% of the total IMV protein. Notably, this approach could not be used to determine the Mm‐mPPase active‐site concentration because Mm‐mPPase binds AMDP much weaker.

### 2.2 Transient kinetics of PP_i_ hydrolysis

**FIGURE 2:**
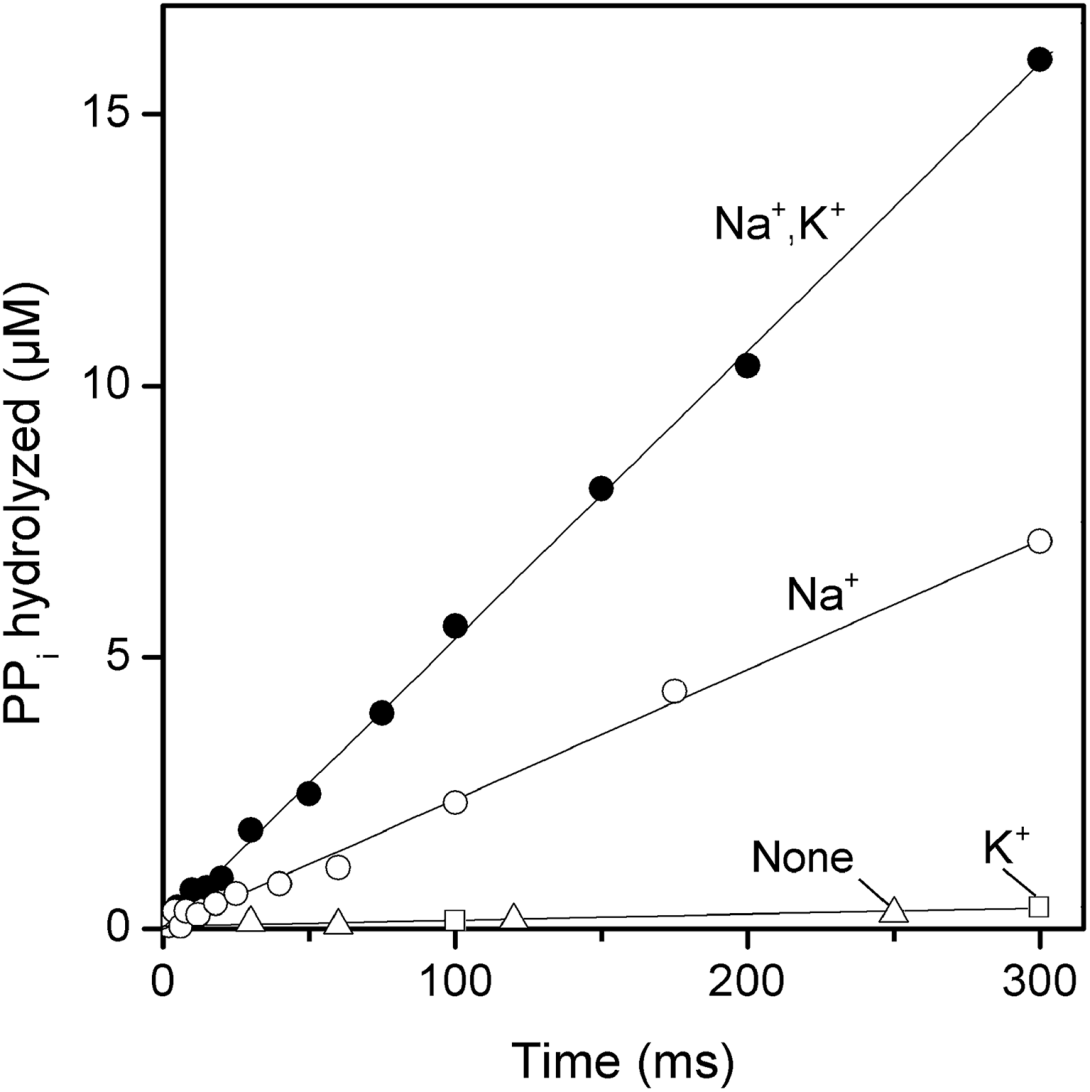
Pre‐steady‐state kinetics of Tm‐mPPase. The enzyme was rapidly mixed with ^32^PP_i_ to yield 8.6 mg ml^−1^ IMV protein (4.3 μM Tm‐mPPase dimer) and 79 μM PP_i_ final concentrations. The hydrolysis reaction was allowed to proceed at 40°C for the time indicated on the abscissa, the mixture was quenched with acid, and ^32^P_i_ in the system was measured. The curve labels refer to the alkali cations present (none, K^+^ only, Na^+^ only, or both Na^+^ and K^+^). Typical results are shown. IMV, inverted membrane vesicle

The first catalytic cycle of Tm‐mPPase was investigated by mixing the enzyme with nearly saturating concentrations of Mg_2_PP_i_ and Mg^2+^, quenching the enzymatic reaction after a short time, and determining the product P_i_. Notably, the P_i_ amount measured in these experiments refers to the sum of enzyme‐bound and medium P_i_ because the acid‐quenching step releases enzyme‐bound P_i_ into the solution. The measured time‐course of P_i_ accumulation was linear and extrapolated to origin in the presence of either Na^+^ as the sole alkali metal activator or both Na^+^ and K^+^ (Figure 2). These findings identified PP_i_ cleavage as the rate‐limiting step in catalysis in both cases. Rate‐limiting product release or conformational change in the enzyme‐substrate complex would result in a product burst (up to 4.3 μM PP_i_ hydrolyzed) or lag, respectively.

The slopes in Figure 2 yield turnover numbers of 5.3 and 12 s^−1^ for Na^+^‐ and Na^+^, K^+^‐activated Tm‐mPPase, respectively. There was no significant product formation in the absence of Na^+^ (Figure 2), suggesting that Na^+^ binds before the chemical step in catalysis.

### 2.3 Pulse‐chase measurements of PP_i_ binding and its Na^+^ requirement

Substrate binding to Tm‐mPPase in the absence of added Na^+^ ions was measured under single turnover conditions. The enzyme was mixed with a 2.5‐fold excess of ^32^PP_i_ in buffer containing only Mg^2+^ or both Mg^2+^ and K^+^ as metal cofactors in this experiment. The ^32^PP_i_‐binding reaction was allowed to proceed for a varied time before diluting with a large excess of nonlabeled PP_i_ solution, which contained Na^+^ (20 mM final concentration) to allow bound PP_i_ hydrolysis (Na^+^ is absolutely required for Tm‐mPPase activity^29^). The mixture was further incubated for 1 s to complete the first reaction cycle, and the amount of bound ^32^PP_i_ hydrolyzed was estimated by measuring the amount of ^32^P_i_ formed. This ^32^P_i_ predominantly arose from enzyme‐bound ^32^PP_i_, considering the negligible probability of medium ^32^PP_i_ binding and hydrolysis after extensive dilution with nonlabeled PP_i_. Based on the data in Figure 2, one can calculate the percentage of medium PP_i_ conversion of <0.7% during the 1 s chase step. However, such hydrolysis of the medium ^32^PP_i_ was evident with longer incubation times with nonlabeled PP_i_ and eventually resulted in a complete conversion of ^32^PP_i_ into ^32^P_i_. One should also keep in mind that some enzyme‐bound ^32^PP_i_ could be released intact as a reversal of the binding reaction during the chase step, causing an underestimation of the amount of the bound PP_i_. In contrast, partial hydrolysis of the bound PP_i_ during the preceding incubation step would result in an overestimation of bound PP_i_, but such hydrolysis does not occur in the absence of Na^+^, according to Figure 2.

**FIGURE 3:**
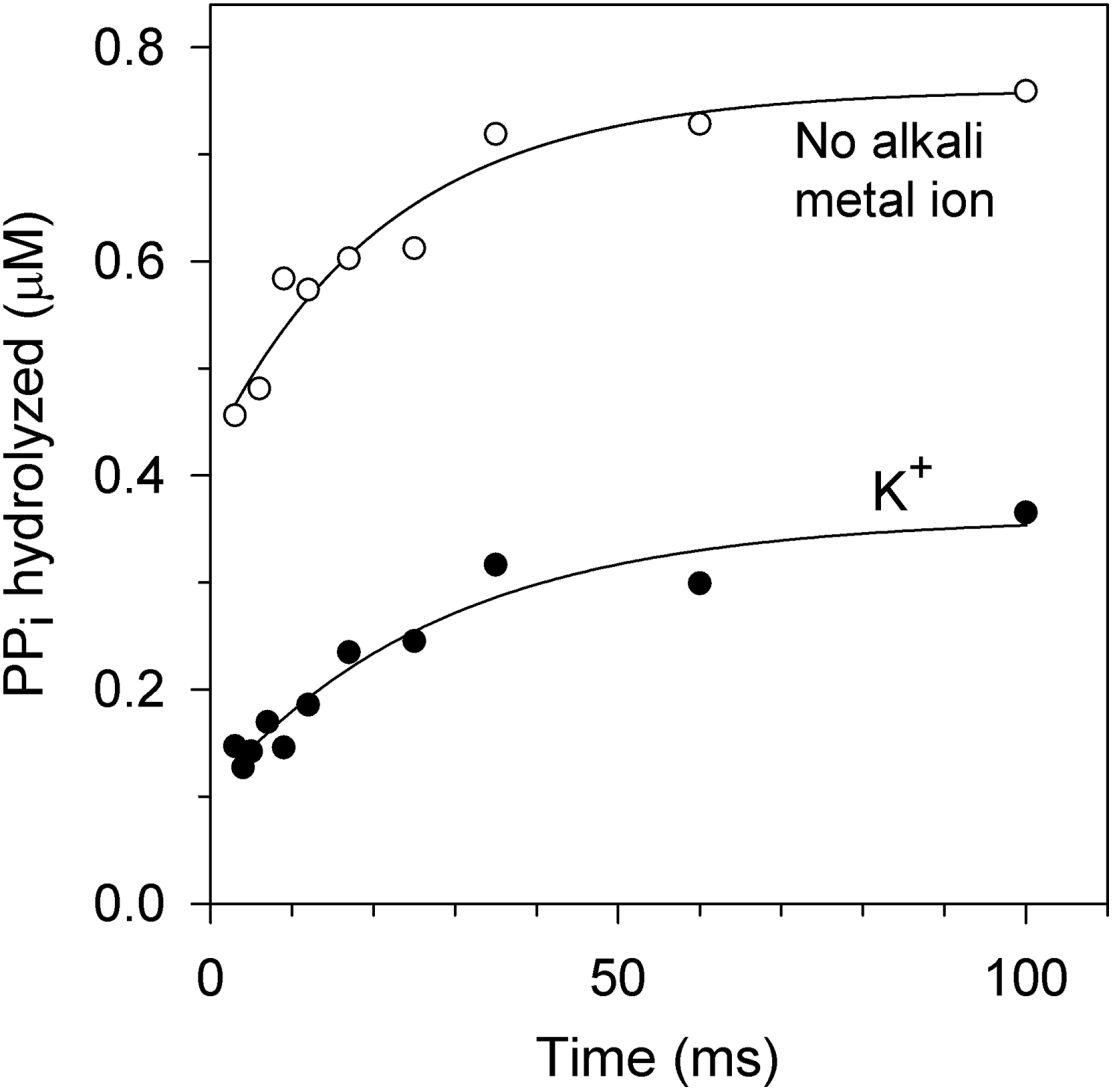
Pulse‐chase measurements of ^32^PP_i_ binding to Tm‐mPPase in the absence of Na^+^. The enzyme was rapidly mixed with ^32^PP_i_ to yield 0.8 μM Tm‐mPPase and 2.5 μM PP_i_ final concentrations, and the binding reaction was allowed to proceed at 40°C for the indicated time before it was arrested by adding an excess of nonlabeled PP_i_ (450 μM final concentration) and NaCl. After 1 s, the mixture was finally quenched with acid, and the amount of ^32^P_i_ in the system was measured. The curve labels indicate whether 50 mM K^+^ was present during the initial binding step. Typical results are shown

One significant finding of these experiments was that ^32^P_i_ formation and, hence, ^32^PP_i_ binding were observed in the absence of Na^+^ during the first incubation step (Figure 3), indicating that this cation is not required for substrate binding. The other significant finding was that the time courses shown in Figure 3 were biphasic, with the first phase unresolved in time by the current technique. Notably, the slower phase in Figure 3 terminated before all PP_i_ was processed and therefore was not associated with an irreversible conversion of PP_i_ into P_i_, insofar as such conversion does not occur in the absence of Na^+^ (Figure 2). Fitting a simple exponent to the slower phase in Figure 3 yielded similar apparent rate constants of 46 ± 10 and 36 ± 10 s^−1^ and different ordinate intercepts—0.33 ± 0.03 and 0.10 ± 0.02 μM for the reaction in the absence and presence of K^+^, respectively. The rate constants exceeded the respective turnover numbers of 5.3 and 12 s^−1^ derived above from Figure 2. The slower phase may thus refer to a relatively rapid conformational change preceding hydrolysis.

K^+^ decreased the amount of ^32^P_i_ formed (Figure 3). This effect could result from decreased ^32^PP_i_ binding, consistent with the earlier finding that K^+^ increases the Michaelis constant for the substrate in several mPPases.^21^, ^36^

### 2.4 Solvent isotope effects on catalysis

If the rate‐limiting step of an enzyme‐catalyzed reaction involves H^+^ transfer, replacing H_2_O with D_2_O as a solvent decreases the observed reaction rate.^40^ Since the effects are generally moderate, the conditions for solvent isotope effect (SIE) measurements with mPPase were chosen to minimize the nonspecific effects of D_2_O on enzyme activity. First, the PP_i_ concentrations used substantially exceeded the respective *K*_m1_ values but were less than *K*_m2_, permitting saturation of only one active site per dimer (“unisite” catalysis). Notably, the values of *K*_m1_ and the Na^+^‐binding constant governing mPPase activation did not vary significantly between H_2_O and D_2_O. Second, Mg^2+^ and alkali metal ions were used at their saturating concentrations. Finally, since pD = pH_read_ + 0.4,^41^ where pH_read_ is the pH meter reading, SIE analysis was performed at a fixed pH_read_ value slightly above the lower boundary of the range in which the rate of the hydrolytic reaction (*V*_1_) was insensitive to pH_read_ in either H_2_O or D_2_O. This setup eliminated complications from an equilibrium isotope effect on a catalytic residue or buffer p*K*_a_. For instance, measurements with Mm‐mPPase were performed at pH_read_ 7.2 because its activity did not vary in the pH_read_ range 6.8–8.0. The pH dependencies of *V*_1_ for other mPPases are shifted to lower or higher pH values; therefore, experiments with them were conducted at different pH_read_ values.

**FIGURE 4:**
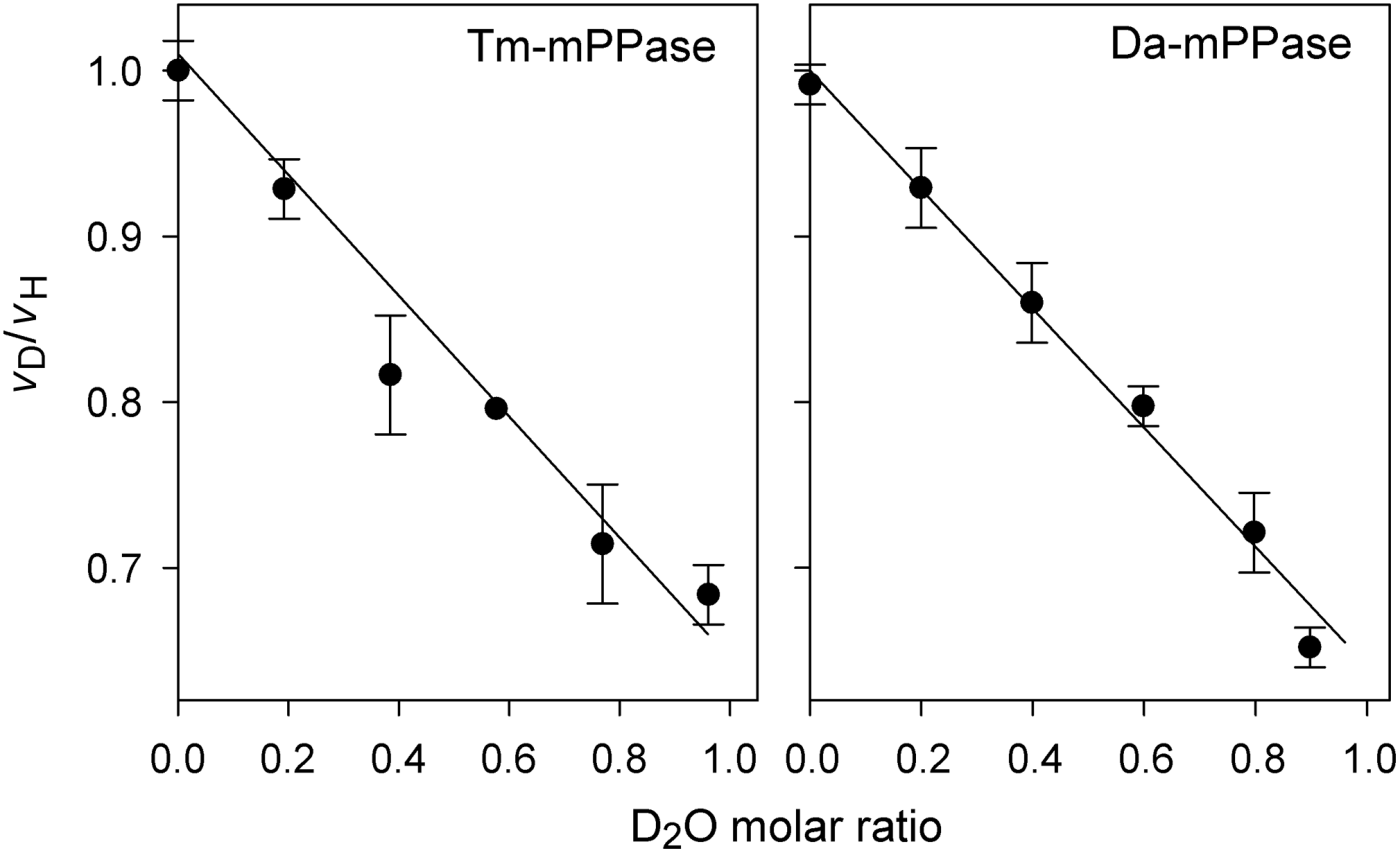
The proton inventory dependence of the Na^+^‐transporting Tm‐mPPase and Da‐mPPase measured under “unisite” conditions. The ordinate shows the ratio of the *V*_1_ values measured in the D_2_O/H_2_O‐ and exclusively H_2_O‐containing media. Bars refer to standard deviations obtained in triplicate measurements. Activity measured in H_2_O was taken as 100%. The lines show the best fit of Equation (4). The assay conditions are listed in Table 2

As Figure 4 highlights, 96% D_2_O decreased *V*_1_ for Tm‐mPPase and *Desulfuromonas acetoxidans* mPPase (Da‐mPPase) by 32 and 35%, respectively, suggesting that a step involving proton transfer determines the overall catalytic rate. Furthermore, the linearity of the rate dependence on the D_2_O molar fraction (*n*) suggested a single proton transfer.^42^ Fitting a modified Gross‐Butler equation for one‐proton inventory (Equation 4) to these dependencies yielded isotopic fractionation factors *ϕ* of 0.66 and 0.64 for Na^+^, K^+^‐activated Tm‐mPPase and Da‐mPPase, respectively. Similar *ϕ* values were obtained for three other mPPases of different transport specificities (Table 2), indicating that the formation of the rate‐determining transition state involves proton transfer in all mPPase types.

(4) $$\frac{v_{D}}{v_{H}}=1+n\left(\phi -1\right),$$

**TABLE 2:** Kinetic solvent isotope effects in PP_i_ hydrolysis by mPPases

Enzyme	Transport specificity	Assay conditions	*V*_H_, nmol·min^−1^·mg^−1^	*ϕ*
Tm‐mPPase	Na^+^	pH_read_ 6.2, 50 mM K^+^, 10 mM Na^+^	320 ± 10	0.66 ± 0.02
Da‐mPPase	Na^+^	pH_read_ 8.5, 100 mM K^+^, 10 mM Na^+^	360 ± 10	0.64 ± 0.01
Mm‐mPPase	Na^+^	pH_read_ 7.2, 100 mM K^+^, 10 mM Na^+^	1,400 ± 20	0.66 ± 0.01
Bv‐mPPase	Na^+^ and H^+^	pH_read_ 6.7, 50 mM K^+^, 10 mM Na^+^	108 ± 1	0.59 ± 0.03
Dh‐mPPase	H^+^	pH_read_ 6.7, 50 mM K^+^	950 ± 10	0.67 ± 0.01

## 3 DISCUSSION

One must answer three key questions to formulate the mPPase transport mechanism: (a) At which step of the hydrolysis reaction is PP_i_ energy input to drive ion transfer against its electrochemical potential gradient in the membrane? (b) What is the origin of the transported H^+^ ion in H^+^‐PPase and Na^+^‐PPases acting as H^+^ transporters at low (<5 mM) Na^+^ concentrations? (c) How is the transport specificity of Na^+^‐PPase modulated by Na^+^? The results of this study provide the answer to the first question and allow informed speculations concerning the remaining questions.

### 3.1 The energy‐coupling step in Na^+^‐transporting and H^+^‐transporting mPPases

One of the hypothetical mechanisms proposed for mPPase (“binding‐change” mechanism) posits that cation transport uses substrate‐binding energy.^20^, ^30^, ^31^ If so, cation binding should precede substrate binding, and Na^+^ as the transported cation offers the possibility to test this prediction, which is inaccessible with a proton whose binding is not easily controllable. The data in Figure 3 provide direct evidence that Na^+^ is not required for substrate binding, which means that Na^+^ and substrate bind independently or Na^+^ binding follows substrate binding. Furthermore, Figure 2 indicates that hydrolysis in the enzyme–substrate complex is arrested without Na^+^. Random‐order binding of Mg_2_PP_i_ and the activating Na^+^ ion was evident from steady‐state kinetics of PP_i_ hydrolysis by Mm‐mPPase,^36^ although it was unclear whether the activating Na^+^ ion is the one that is transported. These findings demonstrated that Na^+^ is required for the catalytic, not binding step and supported the transport model implying energy coupling at the rate‐limiting PP_i_ hydrolysis step. The possibility that energy coupling occurs at the product release steps seems less likely because they are not rate‐limiting and, hence, require less energy to occur.

**FIGURE 5:**
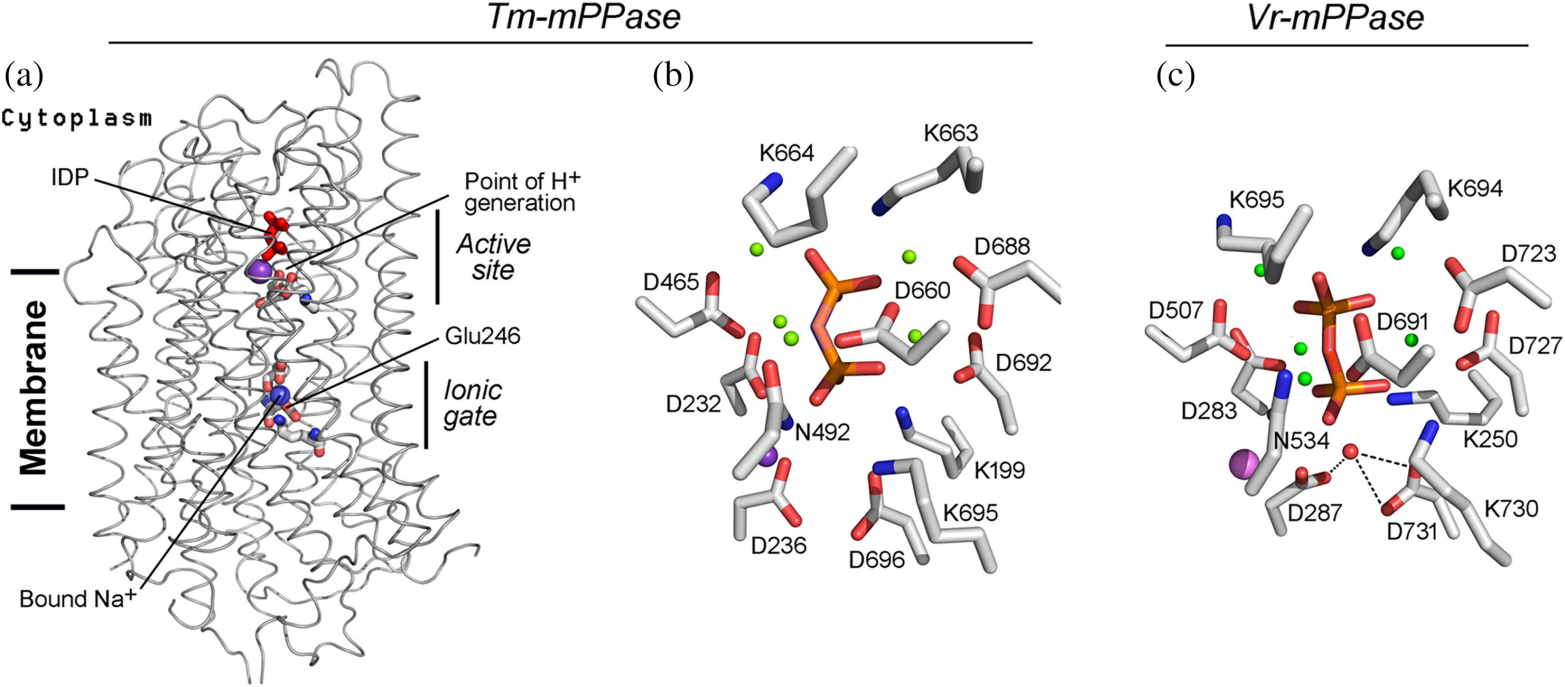
The structures of Na^+^‐ and H^+^‐transporting mPPases. (a), The overall view of *T. maritima* Na^+^‐PPase subunit with bound Na^+^ ion (PDB code: 6qxa; chain B).^22^ (b) The active site of *T. maritima* Na^+^‐PPase with bound imidodiphosphate (red sticks), Mg^2+^ ions (green spheres), and K^+^ ion (magenta sphere). (c), The active site of *V. radiata* H^+^‐PPase (PDB code: 4a01).^19^ The presumed nucleophilic water molecule (red sphere) and its coordination by two Asp residues are shown. Other details are as for panel b

Several lines of evidence suggest that the chemical (hydrolysis) step is likewise the rate‐limiting and coupling step in H^+^‐pumping mPPase, in accord with the notion that H^+^ transfer and PP_i_ hydrolysis occur synchronously in this transporter.^32^ First, similar kinetic isotope effects in Na^+^‐PPases and H^+^‐PPases also point to identical rate‐limiting steps. Second, the active sites of the H^+^‐pumping and Na^+^‐pumping mPPases are very similar and contain identical catalytic residues involved in hydrolysis (Figure 5). Finally, the substrate‐binding regions are also similar in mPPase and soluble PPases, suggesting equally high product release rates. In both PPase types, the principal PP_i_‐binding ligands are three to five bridging metal ions,^43^ consistent with similar (micromolar) *K*_m_ values.

Notably, the structures suggest a much lower efficiency of mPPases at the chemical step because of a different mode of nucleophilic water activation. In soluble PPases, the nucleophile is activated by coordination to two or three divalent metal ions, which convert the water to highly reactive hydroxide.^44^, ^45^ In mPPase, the activating metal ions are replaced in this role by two Asp residues (Figure 5), which polarize the water molecule less efficiently. The slow chemical step clearly explains the 1–3 orders of magnitude lower catalytic constant in mPPases than in soluble PPases.

### 3.2 Proton role in Na^+^ transport

As already mentioned, the nucleophilic water molecule is activated by different mechanisms in membrane PPases (Figure 5) and soluble PPases.^38^, ^44^ There are two main reasons for this. First, although magnesium ions polarize the water molecule more efficiently, their insertion into the charged membrane would involve an energy cost. Second, a milder activation of the water nucleophile by carboxylates, without its conversion to hydroxide, permits proton generation in the catalytic reaction in the right place for its subsequent transfer across the membrane. The water‐borne proton creates high local acidity at the entrance to the ion‐conducting channel, which drives the proton against the gradient of its electrochemical potential. That the transported proton belongs to the water molecule, not the coordinating aspartate before the chemical step is evidenced by the mode of their coordination in the *V. radiata* mPPase complex with IDP^19^ (Figure 5b). To add, the program PropKa, version 3.4^46^ predicted the respective p*K*_a_ values of 3.6–3.7 and 0.5–1.2 for Asp287 and Asp731 carboxylates in this complex, indicating that they are ionized under physiological conditions. The Asp287 oxygen atom may initially accept the water‐born proton and, following tautomerization, direct it to the ion‐conducting channel.

Although the above analysis refers primarily to H^+^‐PPase, the overall structural and functional similarity suggests that it is, at least, in part relevant to Na^+^‐PPases, which transport H^+^ in parallel to Na^+.^ ^33^, ^34^ The ion‐conducting channels are very similar in H^+^‐PPases and Na^+^‐PPases, suggesting a common H^+^ transport mechanism. The principal structural difference between the two mPPase types is that the gate‐forming glutamate residue of Na^+^‐PPases is one helix turn closer to the hydrolytic center than in H^+^‐PPases.^20^ The crystal structure of Tm‐mPPase (Figure 5a) indicates that this residue forms a Na^+^‐binding site that can also bind H^+^ on its way through the channel in Na^+^‐PPase.

Because the sodium ion is not a reaction product, it is transported by a different mechanism. The “billiard” hypothesis^26^ unifies the H^+^ and Na^+^ transport activities by placing the proton originating from the nucleophilic water molecule as the common driver. This assertion is supported by similar kinetic isotope effects in Na^+^‐PPases and H^+^‐PPases (Table 2). The reactions in D_2_O are typically slower because of its lower vibrational zero‐point energy, and hence, a higher activation energy is required to break the O—D bond than the O—H bond. Noteworthy, all the Na^+^‐PPases used in the isotope effect measurements catalyze the transmembrane transfer of Na^+^ but not H^+^ at the 10 mM Na^+^ concentration used.^33^ The isotopic fractionation factor *ϕ* for both types of mPPase is larger than that for soluble yeast PPase^47^ despite similar reaction chemistry. Moreover, the isotope effect in the soluble PPase primarily results from a different step, that is, product release.^47^ Our findings thus emphasize the crucial role of the proton‐generating step in both mPPase types.

**FIGURE 6:**
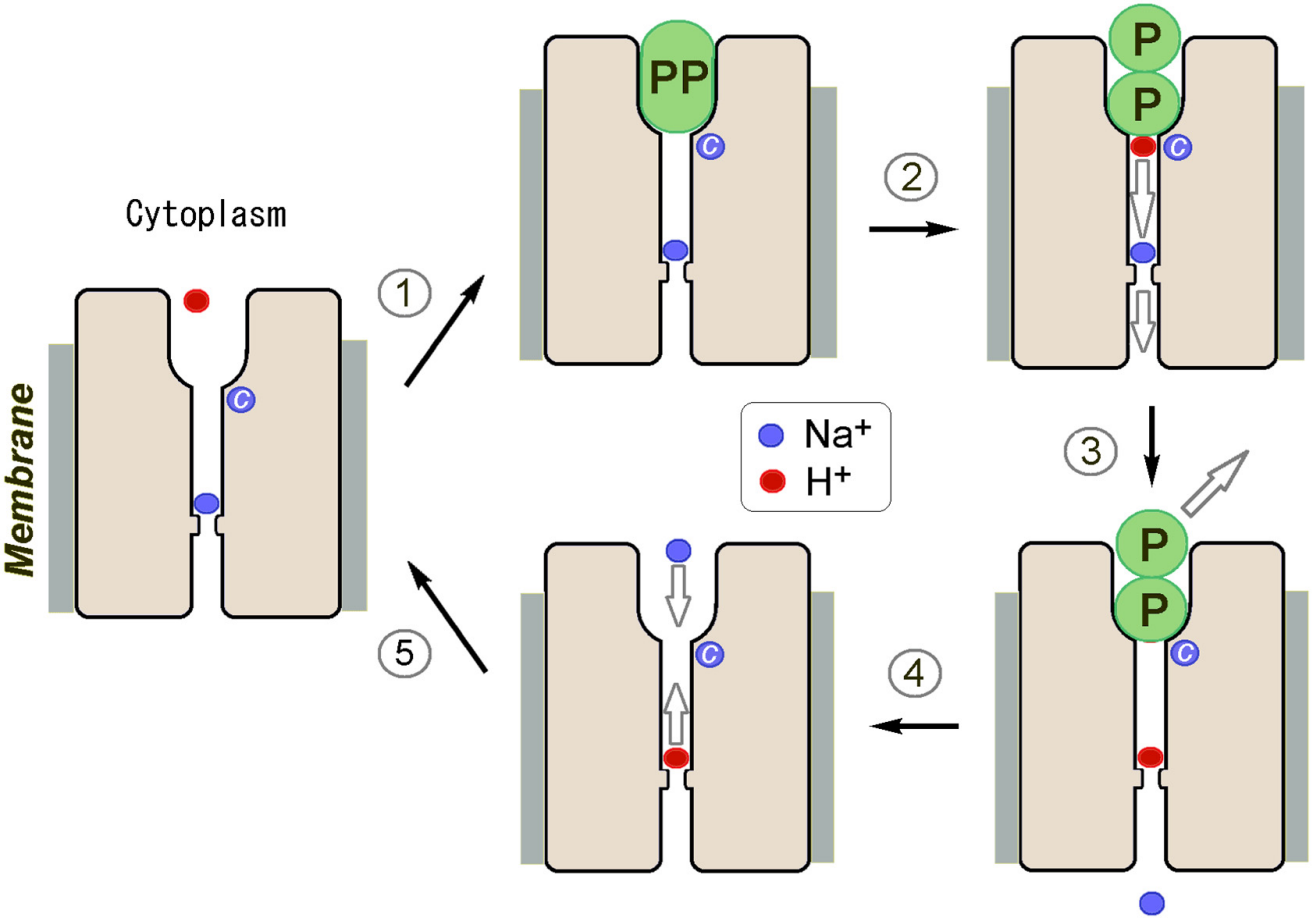
A schematic mechanism of Na^+^ transport by Na^+^‐PPase. Na^+^ ion and proton are shown as blue and red circles, respectively; PP is pyrophosphate and P is phosphate. The Na^+^ ion marked by “*C*” is absolutely required for catalysis and remains permanently bound during the catalytic cycle. Step 2 is rate‐determining. The scheme does not consider the conformational changes accompanying substrate binding and hydrolysis

The available data suggest the minimal H^+^ and Na^+^ transport mechanism in Na^+^‐PPase illustrated in Figure 6. The cation‐binding site formed by the Glu residue near the gate contains a Na^+^ or H^+^ ion in the resting state according to the principle of the local electroneutrality of stable intermediates.^48^ PropKa predicts a p*K*_a_ of 7.0–7.2 for the gate Glu residue (Glu246) of Tm‐mPPase, suggesting its appreciable protonation in the resting state. Functional studies indicated the presence of an additional, Na^+^‐specific, high‐affinity site (marked by “*C*” in Figure 6), whose occupancy by Na^+^ is required for PP_i_ hydrolysis and H^+^/Na^+^ transport.^29^, ^33^ This site appears to be permanently occupied at physiological Na^+^ concentrations. The whole transport reaction at high Na^+^ concentrations (H^+^ transport arrested) may involve five steps in Na^+^‐PPase. (Step 1) The substrate binds, closing the channel on the cytoplasmic side. (Step 2) The hydrolysis reaction commences, releasing a proton at the channel entrance. (Step 3) The proton reaches the gate through linked water molecules (Grotthuss mechanism), forcing the Glu‐bound Na^+^ or H^+^ ion to enter the exit channel and finally be released to the periplasm. (Step 4) Products diffuse from the active site; the Glu residue again becomes protonated. (Step 5) The cytoplasmic Na^+^ partially or wholly replaces the Glu‐bound proton through the open active site, preparing the protein for the next hydrolysis/transport cycle. The kinetic limitation of Na^+^ delivery to its binding site does not seem likely as it takes <20 ms for Na^+^ to cross membrane‐integral Na^+^‐rhodopsin, another Na^+^ transporter.^49^

This tentative mechanism provides a simple explanation for Na^+^ modulation of transport specificity. For this mechanism, the ratio of the Na^+^ and H^+^ transport rates depends on their gate site occupancies, which are determined by their binding affinities and cytoplasmic concentrations. Na^+^ fully occupies the gate site at high concentrations and becomes the only transported cation, redirecting the “chemical” proton to the cytoplasm. At which Na^+^ concentration will the gate Glu bind equal amounts of Na^+^ and H^+^? As already mentioned, the predicted p*K*_a_ of the gate Glu246 in Tm‐mPPase is 7.0–7.2, indicating a 300–500 times lower *K*_a_ value than expected for the Glu carboxylate in an aqueous medium. The dissociation constant of sodium acetate in water is 1.5–1.8 M^50^, ^51^ but will become approximately 7 mM in mPPase if the environment changes the carboxylate affinities for proton and Na^+^ equally. In accord with this calculated value, switching between H^+^ and Na^+^ transport occurs at low millimolar Na^+^ concentrations in most Na^+^‐PPases.^33^ Assuming simple competition between Na^+^ and H^+^ for binding to the gate Glu carboxylate, the ability of some Na^+^‐PPases to transport H^+^ at a 100 mM Na^+^ concentration may result from a higher H^+^‐binding or lower Na^+^‐binding affinity of the gate site.

### 3.3 Kinetic evidence for a conformational change during substrate binding

The biphasic kinetics of substrate binding to mPPase suggests that the enzyme undergoes a conformational change (isomerization) before the hydrolysis step. The rapid binding phase in the kinetic curves in Figure 3 appears to be diffusion‐controlled, based on the value of its rate constant of >10^9^ M^−1^ s^−1^ estimated from Figure 3, and can be assigned to the second‐order reaction of PP_i_ binding. The presumed isomerization reaction can be associated with the slower phase. Notably, the effects seen in Figure 3 do not refer to bound PP_i_ hydrolysis, which does not occur without Na^+^.

**SCHEME 1:**
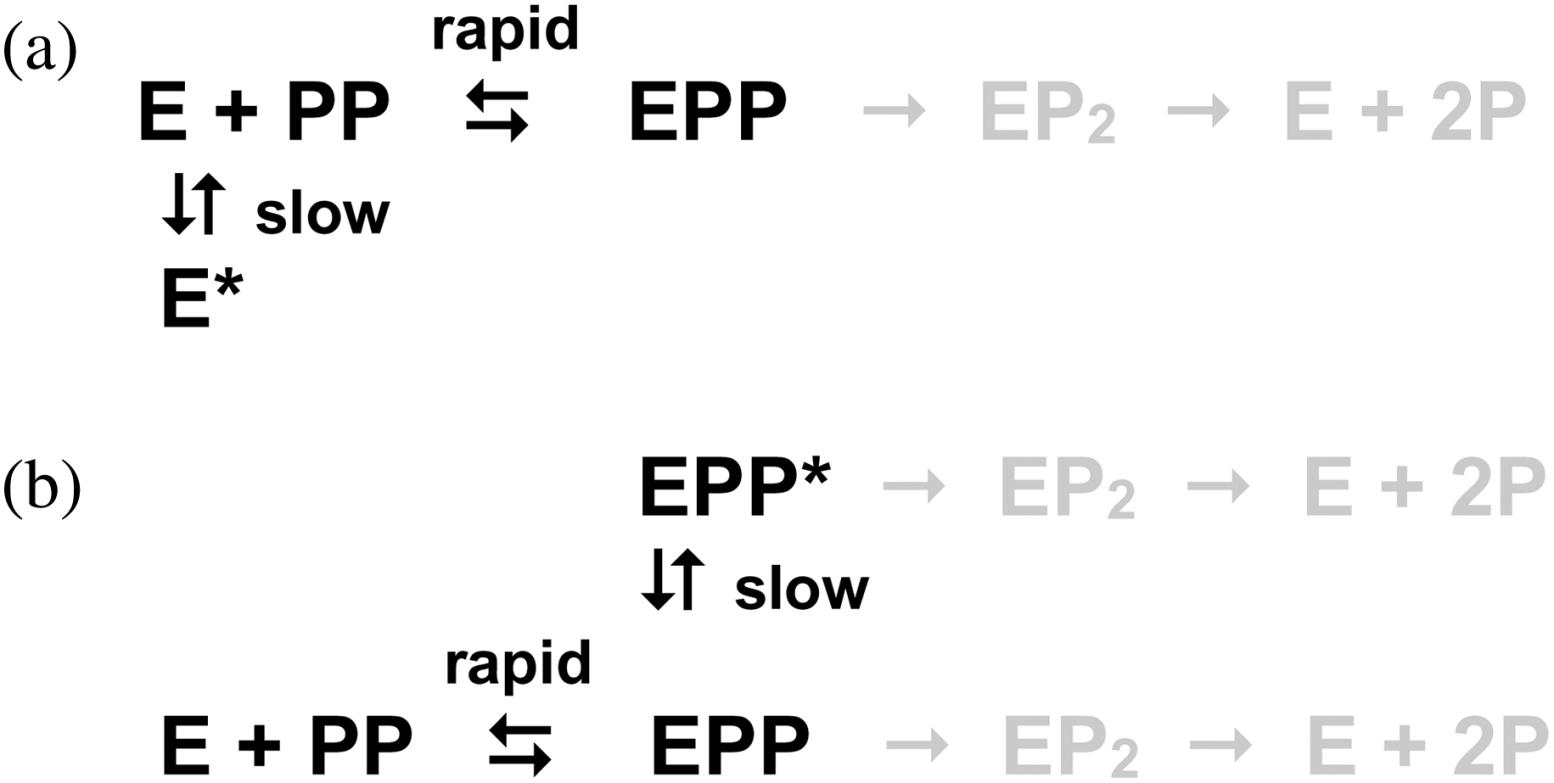
Two kinetic models for PP_i_ hydrolysis by Tm‐mPPase involving enzyme isomerization between two conformations. E is enzyme, PP is pyrophosphate, and P is phosphate; metal cofactors are not shown for simplicity. The asterisk indicates a different conformation. Only the species shown in black are formed in the experiments illustrated in Figure 3

The pulse‐chase data can be interpreted in terms of two kinetic models assuming that the isomerization reaction involves substrate‐free enzyme (Scheme 1a) or the enzyme–substrate complex (Scheme 1b). In model A, there is a preexisting equilibrium between two conformations with essentially different affinities to substrate in the resting enzyme, and this equilibrium is slowly shifted upon rapid substrate binding, producing more enzyme in the binding‐competent conformation. In model B, the isomerization of the enzyme–substrate complex may be a required step of the complete hydrolysis reaction, or a side process, depending on which enzyme–substrate complex, EPP, or EPP*, leads to products. In both models, the isomerization reaction increases the amount of the PP_i_‐bound enzyme, in accordance with the data in Figure 3.

In principle, the presumed isomerization step should cause deviations of the product formation curves from a linear form by causing a product burst. However, large deviations are not expected because the rate constant for the isomerization step, as estimated from Figure 3, exceeds *k*_cat_. A more detailed study is, however, needed to determine the isomerization rate constant in the presence of Na^+^ for a direct comparison with *k*_cat_.

A plethora of data obtained by single‐molecule fluorescence measurements,^52^ kinetic analysis of protection against proteolysis and chemical modification,^36^ X‐ray crystallography,^30^ and molecular dynamics simulations^53^ indicated a conformational change in different Na^+^‐PPases induced by substrate or its analog binding. To the best of our knowledge, our study provided the first demonstration of two conformations of the same enzyme species (presumably, the enzyme−substrate complex). Finally, although our data refer to Na^+^‐PPase, a similar conformational change may occur in H^+^‐PPase.

## 4 MATERIALS AND METHODS

### 4.1 Reagents

Tetramethylammonium (TMA) PP_i_ was prepared by passing a solution of tetrasodium PP_i_ through a Dowex 50 W X8 (Serva) column of charged with TMA^+^. PP_i_ concentration in the eluate was determined by measuring the amount of P_i_ after boiling with 1 M hydrochloric acid to hydrolyze PP_i_. ^32^PP_i_ was obtained from Perkin Elmer, D_2_O from Cambridge Isotope Laboratories, buffer components and most other chemicals from Sigma–Aldrich.

### 4.2 Protein preparation

Na^+^‐transporting mPPases from *T. maritima*,^29^ *M. mazei*,^3^ *D. acetoxidans*,^33^ *Bacteroides vulgatus*,^34^ and H^+^‐transporting mPPase from *Desulfitobacterium hafniense* ^21^ were produced in *Escherichia coli* C41(DE3) cells. IMVs were prepared by a French press method and isolated using a three‐step ultracentrifugation procedure.^3^, ^29^ The membrane pellet was suspended to 15–25 mg ml^−1^ in storage buffer (10 mM MOPS‐TMA hydroxide, pH 7.2, 150 mM sucrose, 1 mM MgCl_2_, and 40 μM EGTA), frozen in liquid‐N_2_, and stored at −70°C. The Tm‐mPPase used in the pH studies was partially purified by heating the IMV suspension to 70°C and sedimenting the precipitate formed in a microcentrifuge. This additional treatment yielded 30–50% pure Tm‐mPPase with a complete recovery of activity.^25^ IMV and partially purified Tm‐mPPase were quantified according to their protein content, which was estimated using the Bradford assay.^54^

### 4.3 Activity measurements

mPPase activity was assayed by following P_i_ production from PP_i_ using an automatic P_i_ analyzer^35^ at a sensitivity of 2–4 μM P_i_ per recorder scale. The reaction mixture typically contained 100 mM MOPS‐KOH or MOPS‐NaOH buffer, pH 7.2. The concentrations of MgCl_2_, KCl, and NaCl are found in the descriptions of the particular experiments. The reaction was started by the addition of PP_i_ (tetrasodium salt), and P_i_ liberation was continuously recorded for 3–4 min. When significant deviations from linearity were observed, initial velocities were estimated from analyzer recordings as described previously.^35^ The concentrations of Mg_2_PP_i_ (the actual substrate) and free Mg^2+^ ions in the assay medium were maintained as described in the same publication. The rates of hydrolysis (mPPase activities) are given below in terms of the amount of total PP_i_ hydrolyzed per 1 min.

### 4.4 Active site titration

Tm‐mPPase‐containing IMVs (0.5 mg total protein) were preincubated for 1 min with 0–50 nM tightly binding PP_i_ analog, AMDP, in 25 ml of 50 mM MOPS‐TMA hydroxide, pH 7.2, 5 mM MgCl_2_, and 100 mM NaCl at 25°C. PP_i_ (8 μM) was added, and the enzymatic reaction was continuously monitored with the automatic P_i_ analyzer. The active‐site concentration was determined from the dependence of activity on AMDP concentration.

### 4.5 Quenched‐flow measurements

Equal volumes (14 μl each) of IMV suspension containing 12–20 mg ml^−1^ total protein (6–10 μM Tm‐mPPase) and 158 μM tetrasodium PP_i_ solution (including ~0.03 μCi ^32^PP_i_) were mixed in an RQF‐3 Quench‐Flow Instrument (KinTek) at 40°C. The medium for both reactants was buffer Q (100 mM MOPS‐TMA hydroxide, pH 7.2, 5.2 mM MgCl_2_, 50 μM EGTA) supplemented with either 100 mM NaCl or 10 mM NaCl and 50 mM KCl. The final concentration of PP_i_ after mixing, 79 μM, corresponded to 50 μM Mg_2_PP_i_ complex (assumed true substrate).^35^ After 5–1,000 ms, the reaction was terminated with 85 μl of 1 M hydrochloric acid. The product mixture was collected into a microcentrifuge tube containing 530 μl of 1.8 mM KH_2_PO_4_ and stored on ice until the whole kinetic curve samples were acquired. After removing the precipitated protein by centrifugation (5 min, 14,000*g*), the supernatants were transferred to new tubes containing 25 mg of glass beads (150–212 μm, Sigma), added to facilitate the subsequent washing steps. ^32^P_i_ was precipitated from the product mix as a P_i_:molybdate:triethylamine complex^55^ by adding 200 μl of 20 mM ammonium molybdate and 50 μl of 100 mM triethylamine‐HCl, pH 5.0. After 5 min, the yellow P_i_ precipitate was isolated by centrifugation (8,000*g*, 5 min). Residual ^32^PP_i_ was removed from the pellet by resuspension in 800 μl of washing solution (prepared by combining 1 volume of 100 mM triethylamine‐HCl, pH 5.0, 1.6 volumes of 1 M hydrochloric acid, 4 volumes of 20 mM ammonium molybdate, and 10.8 volumes of water), followed by centrifugation; the resuspension/centrifugation cycle was repeated three times. The final P_i_ sediment was dissolved in 100 μl of 1 M aqueous ammonia, 1 ml of Ultima Gold cocktail (Perkin Elmer) was added, and ^32^P_i_ was counted by liquid scintillation. Control experiments indicated ~95% recovery of ^32^P_i_, while the amount of co‐purified ^32^PP_i_ was negligible. Appropriate corrections were made for contaminating ^32^P_i_ in ^32^PP_i_ stocks (2–4%, depending on the batch).

For ^32^PP_i_ pulse‐chase experiments, the Quench‐Flow Instrument was supplemented with a syringe charged with 600 μM nonlabeled tetrasodium PP_i_ and 25 mM NaCl. Equal volumes (14 μl each) of 1.6–2 μM Tm‐mPPase and 5 μM ^32^PP_i_ solutions in buffer Q with or without 50 mM KCl were mixed and allowed to react for 5–150 ms before mixing with 85 μl of 600 μM nonlabeled PP_i_. The mixture was further incubated for 1 s to hydrolyze enzyme‐bound ^32^PP_i_, quenched with hydrochloric acid, and processed as described above.

### 4.6 Measurement and analysis of solvent isotope effects

The buffers used to measure kinetic SIEs were prepared in H_2_O or D_2_O and contained 100 mM MOPS, 20 mM MgCl_2_, 0.1 mM EGTA, and varying concentrations of NaCl and KCl. The buffers were adjusted to pH_read_ values of 6.2 (Tm‐mPPase), 7.2 (Mm‐mPPase), or 6.7 (Dh‐mPPase and Bv‐mPPase) with concentrated KOH or NaOH solutions in H_2_O or D_2_O. TAPS replaced MOPS in the pH 8.5 buffer (Da‐mPPase). The components of the D_2_O buffers were dissolved in D_2_O and lyophilized prior to use. Proton inventories were constructed by varying the atom fraction of D_2_O (*n*) from 0 to 0.95. The IMVs were incubated for 2 min in the respective buffer at 40°C (Tm‐mPPase) or 25°C (other mPPases), and the addition of 114 μM PPi started the hydrolysis reaction. The reaction progress curves were linear, signifying no kinetically significant H/D exchange during the measurement. Twofold changes in Na^+^, K^+^, or PP_i_ concentration had negligible effects on the observed rate, signifying that D_2_O did not affect the saturation of the metal and PP_i_ binding sites. Replacing H_2_O with D_2_O did not affect P_i_ analyzer sensitivity.

### 4.7 Isothermal titration calorimetry

Heat production accompanying Mg^2+^ binding with PP_i_ was measured at 25 and 40°C in a VP‐iTC calorimeter (MicroCal). Tetrasodium PP_i_ and MgCl_2_ solutions were prepared in 0.1 M MOPS/KOH buffer (pH 7.2) containing 10 mM NaCl. Titrations were performed by successive 10‐ or 20‐μl injections of 30 mM MgCl_2_ solution into 1.4 ml of 0.5 mM PP_i_ at 25°C or 0.3 mM PP_i_ at 40°C; the interval between injections was 5 min. Measured heat values were corrected for ligand dilution effects.

## 5 CONCLUSIONS

Dual Na^+^/H^+^ transport specificity is not uncommon among cation transporters, but all such transporters use indirect coupling mechanisms with easy switching between the cations, both of which come from the solution and cross the membrane independently. mPPase uses a direct‐coupling mechanism to pump proton, the product of the hydrolysis reaction, but an indirect‐coupling mechanism to pump Na^+^ ion, which comes from the cytoplasm. This unique combination of two alternative mechanisms has never been observed for any cation transporter. The direct‐coupling H^+^ transport mechanism emerged straightforwardly from the transporter structure,^19^ making H^+^‐PPase the first example of a non‐oxidoreductase proton transporter with such a mechanism.^56^ The question is how this mechanism coexists with indirectly coupled Na^+^ transport. The principal outcome of the current study consists of providing the first experimental evidence of a unique solution for Na^+^ transport—the “billiard‐type” mechanism. Specifically, two types of data—pre‐steady‐state kinetics and kinetic isotope effects—demonstrate that the proton‐generating step is rate‐limiting in Na^+^‐PPase catalysis. Pulse‐chase measurements of PP_i_‐binding indicated that Na^+^ binds independently of the substrate. These findings are entirely consistent with the “billiard‐type” mechanism, although they do not prove it unequivocally.

Future studies will test and extend the proposed Na^+^ transport mechanism. One task is to determine how and in which form the Na^+^ ion passes the ion‐conducting channel. Are there intermediate Na^+^‐binding sites forming a “Na^+^ wire,” similar to the proton wire, between the cytoplasm and the gate? Significant functional and structural asymmetry of the mPPase dimer^21^, ^22^, ^23^, ^53^ suggests that its subunits may work alternately, which raises the possibility of coordinated Na^+^ and H^+^ transport by different subunits.^30^, ^34^ Concurrent transportation of both Na^+^ and H^+^ in one catalytic cycle^34^ is also conceivable.

**Abbreviations**
AMDP: aminomethylene diphosphonate
Bv‐mPPase: *Bacteroides vulgatus* mPPase
Da‐mPPase: *Desulfuromonas acetoxidans* membrane pyrophosphatase
Dh‐mPPase: *Desulfitobacterium hafniense* membrane pyrophosphatase
EGTA: ethylene glycol‐bis(β‐aminoethyl ether)‐*N*,*N*,*N*′,*N*′‐tetraacetic acid
IMV: inverted membrane vesicles
Mm‐mPPase: *Methanosarcina mazei* membrane pyrophosphatase
mPPase: membrane PPase
P_i_
: inorganic phosphate
PPase: pyrophosphatase
PP_i_
: pyrophosphate
SIE: solvent isotope effect
TMA: tetramethylammonium
Tm‐mPPase: *Thermotoga maritima* membrane pyrophosphatase

## AUTHOR CONTRIBUTIONS

**Anssi Malinen:** Conceptualization (equal); formal analysis (equal); investigation (equal); methodology (equal); validation (equal); writing – original draft (equal); writing – review and editing (equal). **Viktor Anashkin:** Formal analysis (equal); investigation (equal); methodology (equal); validation (equal). **Victor Orlov:** Methodology (equal); resources (equal); validation (equal). **Alexander V. Bogachev:** Methodology (equal); resources (equal); supervision (equal); validation (equal); writing – review and editing (equal). **Reijo Lahti:** Methodology (equal); supervision (equal); validation (equal). **Alexander Baykov:** Conceptualization (equal); formal analysis (equal); methodology (equal); supervision (equal); validation (equal); visualization (equal); writing – original draft (equal); writing – review and editing (equal).

## FUNDING INFORMATION

Anssi M. Malinen was funded by the Academy of Finland grant 307775.

## CONFLICTS OF INTEREST

The authors declare no conflicts of interest.

## Data Availability

The data that support the findings of this study are available from the corresponding author upon reasonable request.

## APPENDIX A

### A.1 STABILITY AND SOLUBILITY OF THE MAGNESIUM–PYROPHOSPHATE COMPLEXES AT ELEVATED TEMPERATURES

Adequate analysis of PPase steady‐state kinetics critically depends on maintaining constant free Mg^2+^ concentration, thereby making substrate (S = Mg_2_PP_i_) concentration a single factor affecting catalysis. This is trivially achieved if [Mg^2+^] >> [S] but requires knowledge of the dissociation constants for the magnesium complexes of PP_i_ if this condition is not satisfied. Such constants are available or can be calculated for a broad pH range at 25°C, the standard temperature for enzyme studies.^35^ However, no such data are available for higher temperatures, hampering the studies of the PPases isolated from thermophilic organisms.

We used isothermal titration calorimetry to determine magnesium pyrophosphate stability at 40 and 25°C. The titration curves (Figure A1) were biphasic and indicated that the binding reaction is slightly exothermic at lower and endothermic at higher Mg:PP_i_ ratios. The dissociation constant for MgPP_i_ did not change with temperature, as for HP_2_O_7_^3−^,^57^ and the dissociation constant for Mg_2_PP_i_ decreased with increasing temperature (Table A1). The sign and size of the temperature effect on Mg^2+^ binding to MgPP_i_, which occurs through only one phosphate group of PP_i,_ ^58^ correlate well with the effect of temperature on H^+^ and Mg^2+^ binding to phosphate.^57^, ^59^ The determined MgPP_i_ and Mg_2_PP_i_ stability constants were used to maintain a constant Mg^2+^ concentration in the experiments measuring the substrate concentration dependence of the hydrolysis rate for Tm‐mPPase at 40°C (Figure 4).

**TABLE A1:** Thermodynamic parameters for Mg^2+^ binding to PP_i_ derived from triplicate ITC titrations

Parameter	Value ± SD	
	25°C	40°C
*K*_1_ (μM)	56 ± 4	56 ± 7
*K*_2_ (μM)	1760 ± 60	320 ± 40
∆*H* ^0^_1_ (kJ Mol^−1^)	−4.9 ± 0.1	−9.2 ± 0.8
∆*H* ^0^_2_ (kJ Mol^−1^)	11.6 ± 0.1	12.8 ± 0.6

The low solubility of Mg_2_PP_i_ sets an upper limit for permissible substrate concentration, hampering the saturation of the second active site in the dimer. In the presence of 5 mM Mg^2+^, precipitation was visually observed within 5 min at >1 mM Mg_2_PP_i_ at 25°C and at >0.8 mM Mg_2_PP_i_ at 40°C (0.1 M MOPS‐KOH buffer, pH 7.2). These data were quantitatively confirmed by using centrifugation to separate the precipitated Mg_2_PP_i_. To this end, the mixtures of tetrasodium PP_i_ and MgCl_2_ in the buffer were preincubated for 4–5 min in 1.5‐ml Eppendorf tubes at 25, 40, or 60°C and immediately spun off for 1 min in a microcentrifuge. The PP_i_ concentration in the clear supernatant was estimated from the amount of P_i_ produced upon complete hydrolysis by *Streptococcus gordonii* soluble PPase.^60^ The results indicated that the highest permissible Mg_2_PP_i_ concentration, at which <10% PP_i_ precipitated under these conditions, was 1.2 mM at 25°C and 0.8 mM at 40°C. At 60°C, precipitation started at a PP_i_ concentration of 0.2 mM in the presence of 6 mM MgCl_2_. However, slight opalescence appeared in the sample containing 1 mM Mg_2_PP_i_ after 30 min at 25°C, and a large precipitate appeared after incubation overnight. The sample containing 0.5 mM Mg_2_PP_i_ became slightly opalescent only after overnight incubation at 25°C. No opalescence was observed in the sample containing 0.3 mM PP_i_ and 2.9 mM MgCl_2_ (the final medium composition in ITC titrations) after incubation at 40°C for 2 hr (duration of the ITC titration).

These findings indicated that Mg_2_PP_i_ is poorly soluble but precipitates slowly from its oversaturated solution on the time scale of the enzyme assay or ITC titration in the concentration ranges used. The following calculations support this conclusion. The literature value for the solubility product of Mg_2_PP_i_ at 25°C is 2.5 × 10^−13^ M^−3.^ ^61^ Based on the dissociation constant of 2.5 × 10^−9^ M^2^ for the Mg_2_P_2_O_7_ complex,^37^ one can calculate that Mg_2_P_2_O_7_ solubility is limited to 10^−4^ M at 25°C. Considering that increasing temperature generally increases solubility, elevated temperatures may affect Mg_2_PP_i_ precipitation in oversaturated solutions by increasing its rate.
